# Lactate point-of-care testing for acidosis: Cross-comparison of two devices with routine laboratory results

**DOI:** 10.1016/j.plabm.2015.12.005

**Published:** 2015-12-24

**Authors:** Remco van Horssen, Teska N. Schuurman, Monique J.M. de Groot, Bernadette S. Jakobs

**Affiliations:** a Department of Clinical Chemistry and Hematology, Elisabeth-TweeSteden Hospital, Tilburg, The Netherlands; b Department of Obstetrics and Gynecology, Elisabeth-TweeSteden Hospital, Tilburg, The Netherlands

**Keywords:** POCT, point-of-care testing, ED, Emergency Department, CCU, Critical Care Unit, CV, coefficient of variation, SD, standard deviation, SEE, Standard Error of Estimate, TEa, Total Allowable Error, Lactate, Point-of-care testing, Blood gas, Fetal acidosis

## Abstract

**Objectives:**

Lactate is a major parameter in medical decision making. During labor, it is an indicator for fetal acidosis and immediate intervention. In the Emergency Department (ED), rapid analysis of lactate/blood gas is crucial for optimal patient care. Our objectives were to cross-compare-for the first time-two point-of-care testing (POCT) lactate devices with routine laboratory results using novel tight precision targets and evaluate different lactate cut-off concentrations to predict metabolic acidosis.

**Design and methods:**

Blood samples from the delivery room (*n*=66) and from the ED (*n*=85) were analyzed on two POCT devices, the StatStrip-Lactate (Nova Biomedical) and the iSTAT-1 (CG4+ cassettes, Abbott), and compared to the routine laboratory analyzer (ABL-735, Radiometer). Lactate concentrations were cross-compared between these analyzers.

**Results:**

The StatStrip correlated well with the ABL-735 (*R*=0.9737) and with the iSTAT-1 (*R*=0.9774) for lactate in umbilical cord blood. Lactate concentrations in ED samples measured on the iSTAT-1 and ABL-735 showed a correlation coefficient of *R*=0.9953. Analytical imprecision was excellent for lactate and pH, while for pO_2_ and pCO_2_ the coefficient of variation was relatively high using the iSTAT-1.

**Conclusion:**

Both POCT devices showed adequate analytical performance to measure lactate. The StatStrip can indicate metabolic acidosis in 1 μl blood and will be implemented at the delivery room.

## 1 Introduction

Lactate is a crucial metabolite produced under stress conditions, like hypoxia. In the absence of oxygen, glucose is converted into lactate, while normally glucose is fully oxidized in the cell mitochondria to efficiently produce energy (adenosine triphosphate, ATP). Intracellular produced lactate is secreted as lactic acid, resulting in metabolic acidosis. In case of severe sepsis, lactate is produced by micro-organisms, as these do not have mitochondria.

Blood lactate is an indicator for metabolic acidosis and can serve as a single marker for immediate medical intervention. In the Emergency Department (ED) it can indicate sepsis and ischemia. In critically ill patients lactate concentrations are reported to correlate well with disease state, have prognostic value and can be used for monitoring [1], [2]. A short turn-around-time for lactate measurements, preferably as a component of full blood gas analysis, will support prompt medical decision making. Measurement by POCT devices can be an adequate method to achieve this, but one should always consider analytical performance, user competences and costs-benefits ratios. In the department of Obstetrics, fetal lactate is a direct indicator of metabolic acidosis and can therefore be used to assess the need for intervention during labor. Currently, along with intra-partum cardiotocography to measure fetal heart rate and uterine activity, fetal scalp blood can be collected to monitor fetal status. Blood tests include fetal pH, combined with other blood gas parameters (base excess, pCO_2_) to identify fetal acidosis [3], [4], [5]. It is necessary to distinguish between respiratory and metabolic acidosis, as poor neonatal outcome is associated with the latter. However, a major drawback is the amount of sample needed to perform full blood gas analysis (40–90 μL), which results in very high failure rates, up to 23% [6], [7]. Since lactate is responsible for metabolic acidosis and, moreover, can be measured in very small volumes of blood (1 μL), direct measurement of lactate is a very attractive alternative. A number of studies have evaluated whether lactate can replace pH, measured in umbilical cord or fetal scalp blood [7], [8], [9], [10]. The common conclusion of these studies is that lactate concentrations represent fetal status equivalently to pH measurement. To evaluate the analytical performance of lactate POCT devices in this setting, several studies have compared lactate concentrations in umbilical cord and fetal scalp blood. Frequently evaluated lactate POCT devices are strip-analyzers, like the Lactate-Pro (Arkray) and StatStrip Lactate (Nova Biomedical), which have been shown to have satisfactory analytical performance. The StatStrip Lactate was found to perform the best, based on interference studies and imprecision (coefficient of variation, CV) measurements [11], [12]. Recently, these conclusions were confirmed in a large study population resulting in a strong reduction in failure rates, compared with fetal scalp blood sampling, mainly caused by a shortage of sample resulting in no measurement on blood gas analyzers [6]. Noticeably, the cut-off value of lactate used to predict fetal metabolic acidosis differs strongly between the studies (5.1 mmol/L versus 6.6 mmol/L), which is primarily explained by the use of different analyzers.. The possibilities of lactate measurement with POCT devices at the bedside of patients in the ED and Critical Care are also explored in the literature [13], [14], [15]. Interestingly, for patients with metabolic acidosis, biological variability of lactate was shown to be about half that of healthy individuals, which is clearly important for validation of lactate POCT devices [16].

In view of the above mentioned clinical settings and recent literature, we set up a study, comparing two POCT devices (which have not previously been compared) with routine blood gas analysis. The StatStrip Lactate and the iSTAT-1 (for full blood gas analysis, including lactate) were compared with a routine blood gas analyzer (ABL-735) and with each other. We also evaluated the published cut-off values for fetal lactate and incorporated the new insights of biological variation of lactate concentrations in critically ill patients.

## 2 Materials and methods

### 2.1 Patient samples

151 blood samples from patients were used, including 66 umbilical cord samples from the Department of Obstetrics and 85 arterial samples from the ED and Critical Care Unit (CCU). This study was done on surplus material and patients who refused re-use of their blood were excluded. According to Dutch legislation covering this kind of validation research, no informed consent and approval of the Medical Ethics Review Board is necessary, and this was confirmed by the local Medical Ethics Review Board of the Elisabeth-TweeSteden Hospital under reference number: METC-Brabant/15.147. This implies that there is no extra handling for subjects, there is no difference in treatment, no difference in analysis, no effect on diagnosis and also no in- or exclusion criteria as in comparative diagnostic studies with control and study groups. For this reason the STARDChecklist was not used as most issues are not applicable to this study [17]. Umbilical cord blood was drawn in the delivery room after the baby was born. Samples from the ED and CCU were collected within the timeframe of the study, after routine analysis was complete. Measurements were performed by laboratory specialists and technicians. All samples were anonymised by removal of the patient identification number.

### 2.2 Comparison of lactate and blood gas measurements

Multiple instrument comparison was performed for the routine blood gas analyzer, ABL-735 (Radiometer, Copenhagen, Denmark) and two POCT devices: the StatStrip-Lactate (Nova Biomedical, Waltham, MA, USA) and the iSTAT-1 (CG4+ cassettes, Abbott Point of Care, Princeton, NJ, USA). All blood samples were measured routinely on the ABL-735. Simultaneously to this routine measurement, lactate concentrations were analyzed in 1 μL blood on the StatStrip. For all blood samples that contained sufficient material, a full blood gas was measured in 90 μL blood on the iSTAT-1 using in the CG4+ cassette. To ensure inclusion of the whole measurement range for lactate, a small subset of blood samples was measured again after 2–6 h. Using this protocol, all parameters in the CG4+ cassette (pH, pCO_2_, pO_2_, base excess, ${\mathrm{HCO}}_{3}^{-}$, sO_2_ and lactate) were measured for 54 umbilical cord blood samples and 77 ED/CCU blood samples on the ABL-735. Lactate concentrations were cross-compared between the two POCT devices and the routine laboratory analyzer for all blood samples.

### 2.3 Precision analysis

Analytical performance of the POCT devices was assessed by measurement of internal quality control material (StatStrip-Lactate Control Solution Level 2 (6.4 mmol/L) (Nova Biomedical, Waltham, MA, USA) and iSTAT-1 TriControls Level 1 (Abbott Point of Care, Princeton, NJ, USA) (Lactate: 7.04 mmol/L; pH: 7.054; pCO_2_: 8.08 kPa; pO_2_: 11.2 kPa). Precision analysis was performed according to the CLSI:EP5A Complex Precision Protocol. Measurements were done twice a day in duplicate for 11 separate days to calculate the within-run, between-run, between-day and total imprecision.

### 2.4 Statistical analysis

Results were statistically analyzed with EP-Evaluator (Version 10.3, David G Rhoads Associates, South Burlington, VT, USA) using the CLSI guidelines: Two-Instruments Comparison (CLSI:EP9), Alternate Method Comparison (CLSI:EP9A), Multiple Instrument Comparison and Complex Precision (CLSI:EP5A). For data plotting and calculation of the bias, Bland–Altman plots were used. To compare two methods and calculate the correlation coefficients (*R*), Passing-Bablok regression analysis was done using EP-Evaluator. The algorithms used include measurement of spreading of the *x*–*y* data around the regression line, to statistically identify outliers. This parameter (Standard Error of Estimates, SEE) is computed from the data set without outliers and an outlier is defined as a point that exceeds 10 time the SEE. If the data set included more than 5% outliers, the method comparison failed. Correlation statistics (*r*^2^) for different parameters (pH and lactate) was performed with Prism Version 4.0 (Graph Pad Software, La Jolla, CA, USA).

Concentration data are expressed as mean±standard deviation (SD).

## 3 Results

### 3.1 Cross-comparison of lactate concentrations

**Fig. 1:**
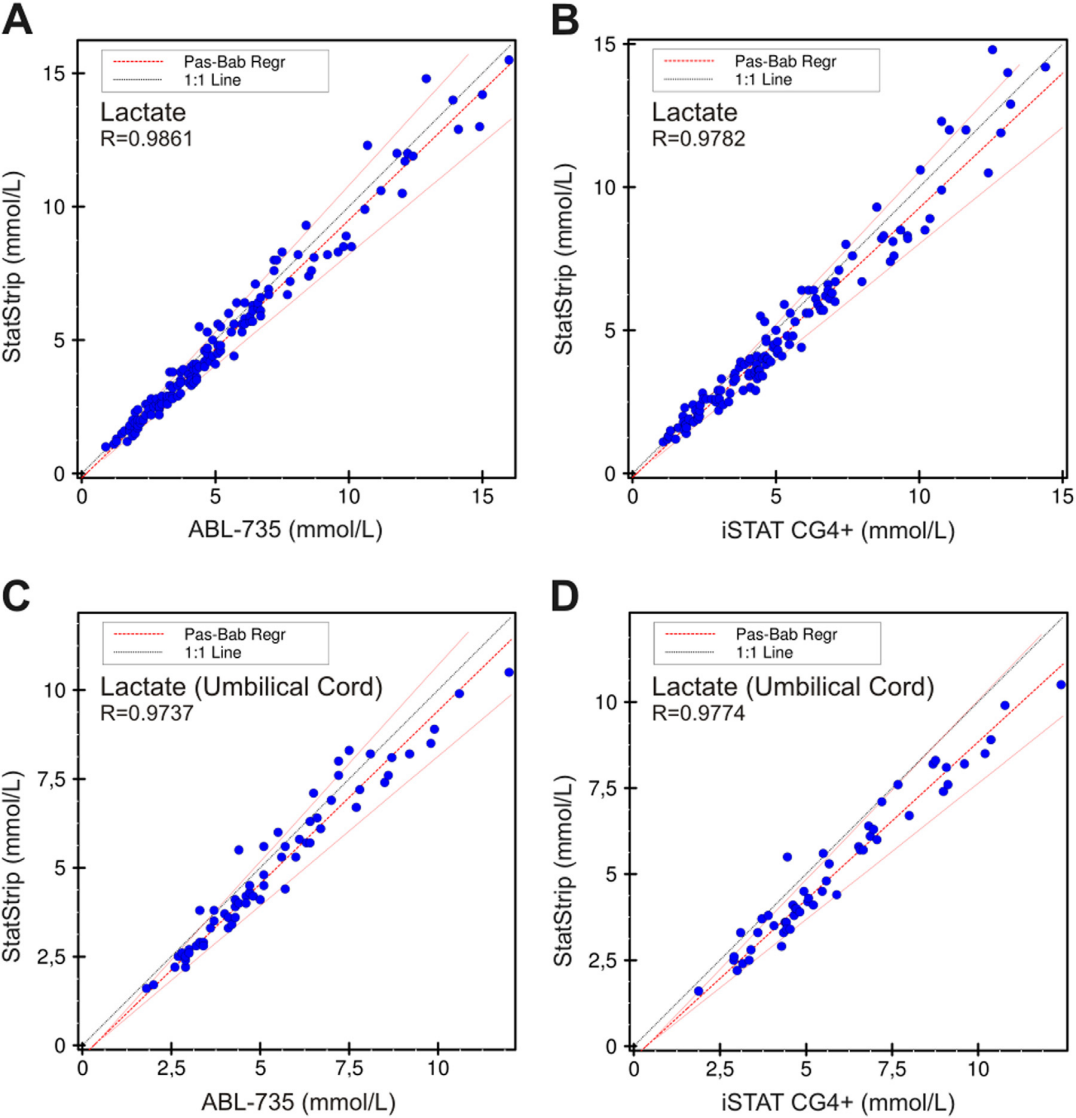
Method comparison for measuring lactate on the StatStrip, the iSTAT-CG4+ and the ABL-735. (A) StatStrip versus ABL-735 for lactate in all blood samples (Bias: -5.3%). (B) StatStrip versus iSTAT-CG4+ for lactate in all blood samples (Bias:-6.84%). (C) StatStrip versus ABL-735 for lactate in umbilical cord blood (Bias: −7.19%). (D) StatStrip versus iSTAT-CG4+ for lactate in umbilical cord blood (Bias: −12.89%).

Lactate concentrations were compared for all blood samples (*n*=151) between the ABL-735 and the StatStrip and for a subset of 131 samples cross-compared between the iSTAT-CG4+ and the StatStrip. The concentration range in the first comparison was 0.9–16.0 mmol/L (ABL-735) and 1.0–15.5 mmol/L (StatStrip) with a mean of 5.2 (±3.2) and 4.9 (±3.2), respectively (Fig. 1A). Cross-comparing the iSTAT-CG4+ to the StatStrip showed concentration ranges of 1.1–14.4 mmol/L and 1.1–14.8 mmol/L with mean values of 5.3 (±3.0) and 4.9 (±3.0), respectively (Fig. 1B). In the umbilical cord blood samples (*n*=66) the same cross-comparison for lactate was performed. The ABL-735 and the StatStrip showed concentration ranges of 1.8–12.0 mmol/L and 1.6–10.5 mmol/L with mean values of 5.4 (±2.2) and 5.0 (±2.2), respectively (Fig. 1C). In 54 umbilical cord blood samples sufficient material was available for cross-comparison of the iSTAT-CG4+ and the StatStrip. These two POCT devices showed aconcentration range of 1.9–12.4 mmol/L with a mean values of 5.8 (±2.4) and 1.6–10.5 mmol/L with a mean of 5.1 (±2.2), respectively.

### 3.2 Comparison of full blood gas parameters of POCT versus routine analysis

**Fig. 2:**
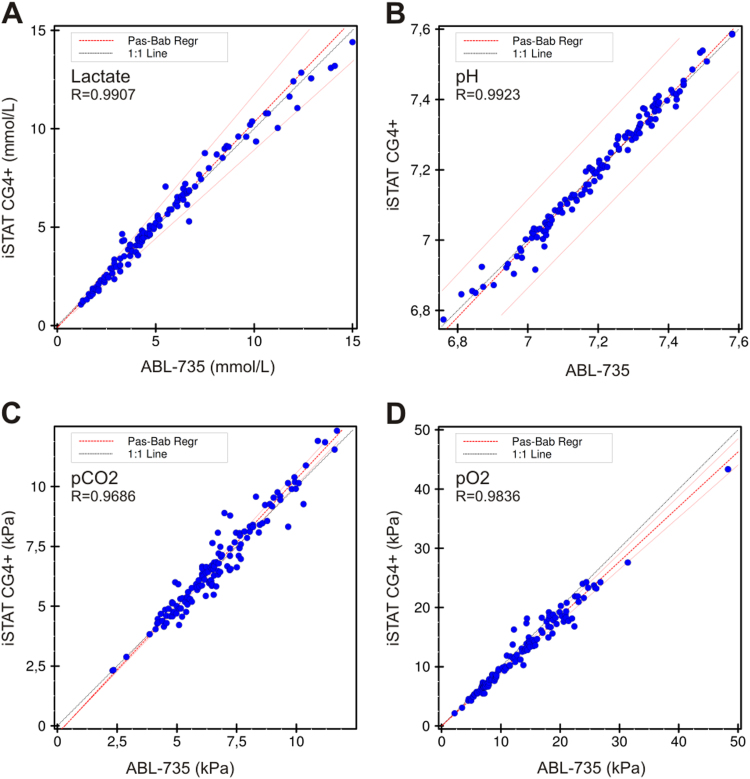
Method comparison for measuring lactate, pH, pCO_2_ and pO_2_ on the iSTAT-CG4+ and the ABL-735 for all blood samples. (A) Lactate, (Bias: 1.6%). (B) pH, (Bias: −0.01%). (C) pCO_2_, (Bias: 0.74%). (D) pO_2_, (Bias: −6.58%). The method comparison for the subset of blood samples of ED/Critical Care patients can be found in Supplementary Fig. 1.

Full blood gas analysis was performed in 131 blood samples, measured routinely on the ABL-735 and with the iSTAT-CG4+. The analyzers were compared only for the measured parameters (lactate, pH, pCO_2_ and pO_2_), excluding the calculated parameters. For lactate we found concentration ranges of 1.2–15.0 mmol/L, mean 5.2 (±3.1) and 1.1–14.4 mmol/L with a mean of 5.2 (±3.0) for the ABL-735 and iSTAT-CG4+, respectively (Fig. 2A). The pH showed a range of 6.76–7.58 with a mean of 7.20 (±0.17) and 6.77–7.59 with a mean of 7.20 (±0.18) for the ABL-735 and iSTAT-CG4+, respectively (Fig. 2B). For pCO_2_ the ranges were 2.3–11.7 kPa and 2.3–12.3 kPa, and for pO_2_ the ranges were 2.2–48.3 kPa and 2.1–43.3 kPa for the ABL-735 and the StatStrip, respectively. Results of these comparisons are presented in Figs. 2C and D. Furthermore, in a subset of blood samples (*n*=77, from the ED/CCU) the same comparison was performed and the results are shown in Supplementary Fig. 1.

### 3.3 Analytical imprecision and bias

Both POCT devices were evaluated for analytical imprecision by comparing the measured CV of internal quality controls with the CV provided by the company, the analytical imprecision and the Total Allowable Error (TEa) [18], [19]. Lactate concentrations, measured with the StatStrip-Lactate, showed a CV of 5.5% (mean: 6.2 mmol/L), which is lower than the CV stated by the company and the target analytical imprecision (13.6%) for lactate, as recently published [16]. Lactate and pH measured with the iSTAT-CG4+ showed very low CV’s of 1.1% (mean: 6.94 mmol/L) and 0.1% (mean: 7.05), respectively. On the other hand, pCO_2_ and pO_2_ measurement showed CVs that were higher than the CVs provided by the company: 3.4% (mean: 8.2 kPa) for pCO_2_ and 6.3% (mean: 12.2 kPa) for pO_2_. The mean bias calculated from the comparison studies showed that the StatStrip measured lactate concentrations 5.3% lower than the ABL-735 while the iSTAT-CG4+ measured lactate 1.6% higher than the ABL-735. For the parameters measured with the iSTAT-CG4+, the biggest mean bias was found for pO_2_: the iSTAT measured 6.6% lower compared to the ABL-735. These results and the other findings on precision and bias are presented in Table 1.

**Table 1:** Quality parameters (all data %) for the StatStrip-Lactate and iSTAT-CG4+ devices. Bias was calculated using the ABL-735 as reference.

	Analytical imprecision^a^	Total Allowable Error (TEa)^a^	CV (Manufacturer)	CV (Measured)	Bias (*vs* ABL-735)

StatStrip-Lactate	13.6	30.4	9.1	5.5	−5.3
iSTAT-CG4+					
Lactate	13.6	30.4	3.7	1.1	1.6
pH	0.1	0.2	0.1	0.1	−0.01
pCO2	2.4	5.7	2.5	3.4	0.7
pO2	4.8	8.0	4.8	6.3	−6.6

a Analytical Imprecision and TEa according to www.westgard.com.

### 3.4 Relation between lactate concentration and pH

**Fig. 3:**
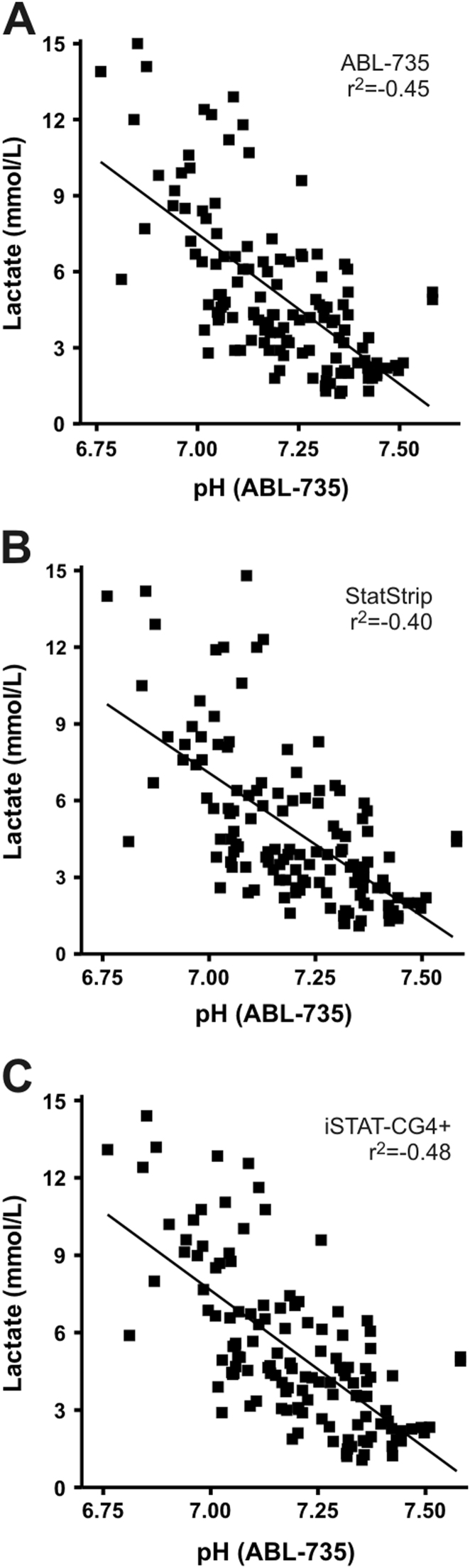
Correlation between lactate and pH for all measured samples. (A) Lactate versus pH, both measured on the ABL-735. (B) Lactate measured on the StatStrip versus pH measured on the ABL-735. (C) Lactate measured on the iSTAT-CG4+ versus pH measured on the ABL-735.

Metabolic acidosis is caused by an increase in lactate. Currently, the pH is used to identify acidotic newborns, but lactate (measured directly with POCT devices) might be suitable to replace pH [8], [10]. Therefore, pH and lactate concentrations were compared, resulting in the following correlations: *r*^2^=−0.45 for the ABL-735, *r*^2^=−0.40 for the StatStrip and *r*^2^=−0.48 for the iSTAT (Figs. 3A–C). These three correlations showed a strong significance (*p*<0.001).

As discussed in the Introduction, the literature contains different lactate cut-off values suggested to predict fetal acidosis [6], [10]. We compared two of the published cut-off values (5.1 and 6.6 mmol/L)For the two POC devices tested for the two POCT devices. The sensitivity and specificity of the lactate cut-off values to predict a metabolic acidosis, represented by a pH of 7.20 (mild) and 7.05 (severe) were calculated. For the StatStrip, a lactate cut-off concentration of >6.6 mmol/L to predict severe acidosis (pH<7.05) showed a sensitivity of 60%, a specificity of 97%, a positive predictive value of 92% and a negative predictive value of 80%. At a lactate cut-off concentration of >5.1 mmol/L a sensitivity of 80%, a specificity of 79%, a positive predictive value of 70% and a negative predictive value of 87% was achieved. For the iSTAT-CG4+, the specificity was lower (88% for lactate>6.6 mmol/L and 71% for lactate>5.1 mmol/L). All test characteristics, including lactate cut-off values for mild acidosis for both POCT devices are presented in Table 2.

**Table 2:** Performance of the StatStrip-Lactate and iSTAT-CG4+ tests to predict acidosis in umbilical cord blood at different Lactate cut-offs and pH levels.

	**Lactate>6.6 mmol/L**		**Lactate>5.1 mmol/L**	
	pH<7.20	pH<7.05	pH<7.20	pH<7.05
*StatStrip-Lactate*				
Sensitivity (%)	25	60	46	80
Specificity (%)	83	97	83	79
PPV^a^ (%)	92	92	96	70
NPV^b^ (%)	12	80	16	87
*iSTAT-CG4+*				
Sensitivity (%)	35	70	52	80
Specificity (%)	83	88	83	71
PPV^a^ (%)	94	78	96	62
NPV^b^ (%)	14	83	18	86

a PPV, positive predictive value.

b NPV, negative predictive value.

## 4 Discussion

Lactate is more specific than pH for prediction of metabolic acidosis c since a low pH can also be caused by other reasons. As well as cardiotocography, fetal scalp blood pH is used to monitor fetal status during labor. Unfortunately, the failure rate is high for these type of blood diagnostics as the the amount of material is often limited and the material may coagulate before analysis [6], [7]. For analysis of lactate by POCT strip analyzers, the amount of material required is only 1 μL, while for pH, either measured on a POCT analyzer or on a blood gas analyzer, much more sample is needed (40–90 μL depending on the device or cassette used). There is growing evidence in the literature that lactate can replace pH to predict metabolic acidosis in both fetal scalp blood and umbilical cord blood [8], [10], [20], [21], [22], [23]. The use of POCT lactate measurements represents an attractive option to obtain a fast result on, most importantly, a small volume of blood. The drawback of a lactate strip analyzer is the fact that lactatealone is measured, while pH measurement is still common practice in many hospitals. The POCT device that meets these criteria, the iSTAT-CG4+, can measure full blood gases including pH and lactate, but does not have the advantage of a small blood volume. Of course, in addition to the practical issues, the analytical performance of the POCT devices must meet the quality requirements as well as routine laboratory blood gas analyzers. In this study the two mentioned POCT devices, StatStrip-Lactate and the iSTAT-CG4+ were – for the first time – cross-compared with routine laboratory results. We used two types of material to ensure we had a range of lactate concentrations. As well as umbilical cord blood we used blood material from the ED and CCU, resulting in lactate concentrations from 1.0–15.5 mmol/L. As all measurements were performed simultaneously, there was no time delay that might affect the outcome of the comparison.

The analytical performance of the StatStrip was good, both for the comparison to routine laboratory results as for the precision analysis. The negative bias we found (versus the ABL-735) was much lower in relation to the analytical CV (5.3% vs 9.1%) and acceptable for clinical practice. In the cross-comparison of lactate measured with the iSTAT-CG4+ we found similar results. In the subgroup of umbilical cord blood we found that the bias was slightly bigger but still acceptable (data not shown). A small negative bias for the StatStrip is in accordance with other accuracy studies for lactate strip analyzers [24]. The analytical performance of the iSTAT-CG4+ was acceptable, and for lactate and pH the correlation and the imprecision were excellent. For pCO_2_ and pO_2_ the iSTAT-CG4+ correlated well with the ABL-735, but the CVs were too high. As these results were still within the total allowable error for these parameters the clinical consequences of this imprecision will be limited [18]. When clinical departments and laboratories consider using the iSTAT-CG4+ this should be taken into account when discrepancies between results appear for a patient measured with the POCT device and on the routine blood gas analyzer. For lactate, as recently published, the imprecision is strongly dependent on the patient population. It was found that for patients with lactic acidosis, the imprecision was about half of that compared to normal individuals [16]. We implemented this tighter precision target and found for both POCT devices that this new target was met.

The correlation between lactate and pH we found was comparable to previous studies [10], [22]. Measurements of acidosis at birth were not included and will be addressed in a follow-up study for clinical validation. The different lactate cut-off concentrations that were tested (5.1 and 6.6 mmol/L), showed 6.6 mmol/L to be the best predictor for a low pH (pH<7.05).Therefore, we propose a test protocol with three decision ranges. Firstly, lactate<5.1 mmol/L indicates absence of acidosis and thus no requirement for intervention. Secondly, when lactate>6.6 mmol/L, this indicates metabolic acidosis of the fetus and thus requires direct intervention. And lastly, an intermediate lactate concentration between 5.1–6.6 mmol/L indicates reassessment of the fetus and repeat of the lactate measurement after 20–30 minutes. For all three cases, when there is sufficient material, pH can be measured during the validation period, but lactate concentrations should be primary. Furthermore, cut-off concentrations should be determined for the specific devices used.

The two POCT devices tested both have practical advantages and disadvantages. A major advantage of the StatStrip is the small volume needed and the simple handling of the device. For the iSTAT, more material is needed, but more parameters are measured in a full blood gas including lactate and pH. On the other hand, the iSTAT is not as user-friendly as the strip analyzers, because more training is required to fill the measuring cassettes. If lactate fully replaces pH for fetal monitoring, the StatStrip is the device of choice. In our hospital the StatStrip will be implemented in a new protocol in the delivery room and will be used either in combination with pH or, when pH measurement fails or is not possible, as a single blood test. In a recent study, new types of blood sample tube are evaluated to reduce the amount of blood sample, as the amount needed for strip analyzers is very low [25]. The iSTAT can only be used in the delivery room when enough material is available. For other clinical settings, such as the ED, where the amount of blood sample is not an issue, the iSTAT-CG4+ blood gas cassette is an attractive option. As is the case for all POCT solutions in hospitals, many aspects need to be taken into account. Next to analytical performance and turn-around-time these include pre- and post-analytical failures, user-friendliness of the instruments, training of non-laboratorians, volume requirements, frequency of testing and last but not least the effects on costs and benefits. In general, POCT analysis is much more expensive compared to analysis in the laboratory, but the above mentioned issues need to be considered in assessing the overall cost-effecitveness. Implementation of new POCT devices is a complex and multifactorial process, with different outcomes in different clinical settings [26].

## 5 Conclusions

Both POCT lactate devices studied showed good analytical performance and met the recently published tight precision target for testing patients with lactate acidosis [16].
