# Supplementary figures and images for: Lactate point-of-care testing for acidosis: Cross-comparison of two devices with routine laboratory results
Source: Pract Lab Med. 2015 Dec 24;4:41–9. doi: 10.1016/j.plabm.2015.12.005 (PMC5574518; doi:10.1016/j.plabm.2015.12.005)

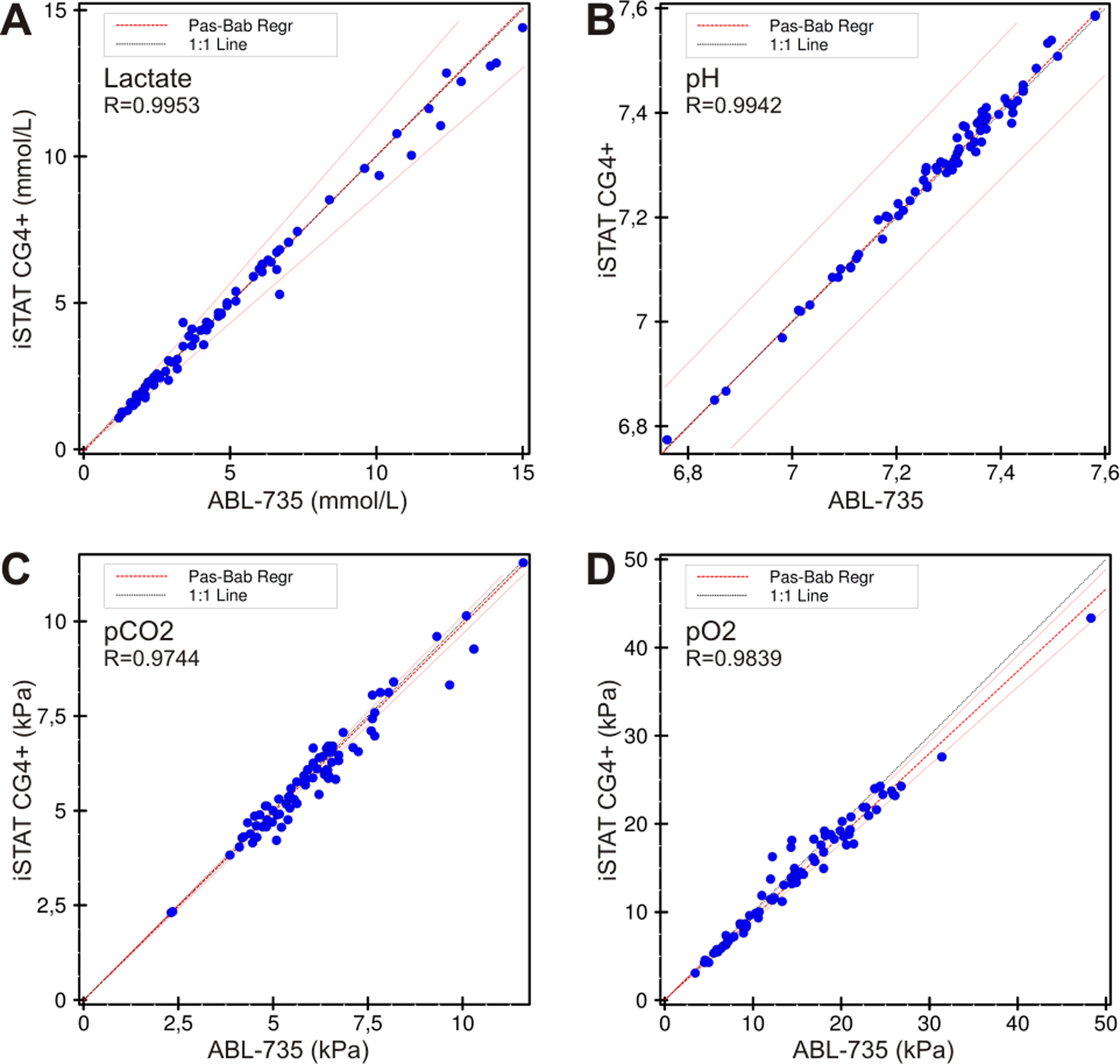

Supplement: Supplementary file 1 — Supplementary material [file mmc1.zip › mmc1.tif]
